# Giant adrenal calcification: rare case report and literature review

**DOI:** 10.1016/j.eucr.2025.103131

**Published:** 2025-07-19

**Authors:** Zaiqing Jiang, Yunfeng Li, Kai Zhao, Xinbao Yin, Ke Wang, Zongliang Zhang

**Affiliations:** a Department of Urology, The Affiliated Hospital of Qingdao University, Qingdao, China; b Department of Immunology and Rheumatology, Qingdao Municipal Hospital, Qingdao, China

**Keywords:** Adrenal gland, Calcification, Case report, Retroperitoneal laparoscopic

## Abstract

We report the largest documented case of idiopathic giant adrenal calcification in a middle-aged woman, incidentally detected during imaging. Preoperative CT revealed an 8.5 × 7.0 × 5.0 cm left adrenal mass. Retroperitoneal laparoscopic adrenalectomy was successfully performed, with no recurrence at 18-month follow-up. This rare condition lacks evidence-based guidelines, necessitating multicenter studies to refine diagnosis and management.

## 1 Introduction

Calcification refers to the pathological process of abnormal calcium salt deposition in necrotic tissues or organs under specific pathological conditions. On imaging studies, calcified lesions typically present as hyperechoic areas with significant acoustic impedance differences (on ultrasonography) or as high-density calcium deposits (on X-ray or CT examinations). Although calcification may occur in various tissues and organs throughout the body, the clinical detection rate of adrenal calcification is significantly lower than that of calcifications at other anatomical sites.^1^ Studies have demonstrated that adrenal calcification may occur secondary to various pathological processes, primarily through two distinct mechanisms:(1) Calcium salt deposition following hemorrhage and necrosis in adrenal tumors; (2) Pathological calcification resulting from primary adrenal disorders of calcium metabolism, such as adrenal hemangioma or adrenal cysts.^1^ Adrenal calcification may also occur secondary to tuberculosis or metastatic malignancies from other systems.^2^ Idiopathic adrenal calcification refers to adrenal calcium deposition lesions of undetermined etiology. Notably, idiopathic giant adrenal calcification is exceptionally rare, with only a few cases reported worldwide. Currently, there remains controversy regarding the necessity of surgical intervention for this condition. It is worth emphasizing that most calcified adrenal lesions exhibit benign pathological characteristics, and the degree of clinical benefit may significantly correlate with the maximum lesion diameter. Strict adherence to surgical indications is crucial to avoid unnecessary medical interventions. This study presents a clinical case of idiopathic massive adrenal calcification.

## 2 Case presentation

**Fig. 1:**
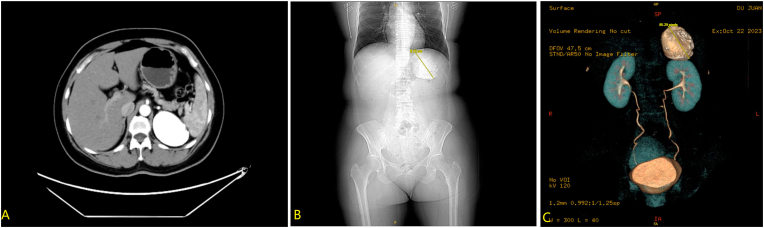
Preoperative CT reveals a calcified lesion in the adrenal region, measuring approximately 8.5 × 7.0 × 5.0 cm, with no enhancement on contrast scan.

A middle-aged female was admitted to our institution following the incidental discovery of a left adrenal mass during routine physical examination one month prior. The patient was completely asymptomatic, reporting no abdominal pain, hypertension, or other complaints. Endocrine workup revealed no abnormalities. Preoperative CT demonstrated an 8.5 × 7.0 × 5.0 cm calcified lesion (Fig. 1). After excluding other potential etiologies, the diagnosis of idiopathic adrenal calcification was established.

**Fig. 2:**
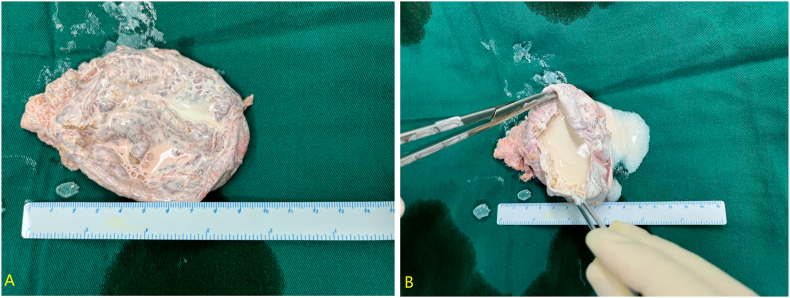
Gross examination of the resected specimen shows a left adrenal mass measuring 9.5 × 6.5 × 4.5 cm. The cyst wall is 0.2–0.5 cm thick, with a smooth serosal surface and a rough inner wall. The cyst contains abundant calcifications and exhibits a hard texture.

**Fig. 3:**
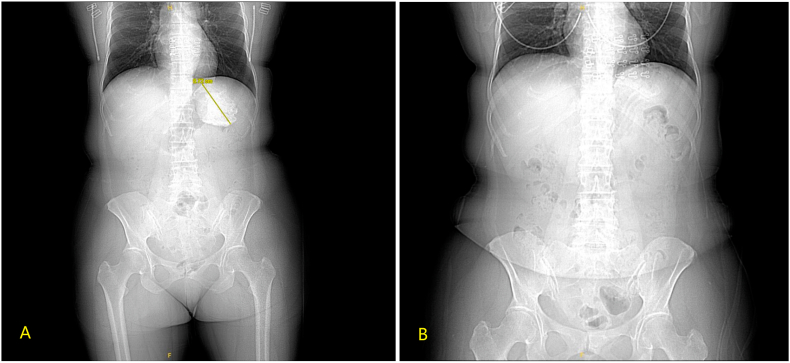
(A) Preoperative imaging demonstrates significant adrenal calcification, whereas (B) postoperative imaging shows no obvious residual calcification.

Given the lesion size exceeding 6 cm in diameter, surgical intervention was indicated. We employed a retroperitoneal approach. Under general anesthesia, the patient was placed in the right lateral decubitus position with the lumbar bridge elevated to optimize exposure of the lumbar space. After establishing the retroperitoneal working space, we carefully dissected the superior pole of the left kidney and medial aspect of the adrenal gland using harmonic scalpel with both blunt and sharp techniques. Intraoperative findings revealed an approximately 8.5 cm whitish calcified mass in the adrenal region with relatively clear demarcation from surrounding tissues. The mass was completely excised after meticulous dissection. Hemostasis was thoroughly achieved, and one plasma drainage tube was placed. The specimen was retrieved using an endobag. The procedure lasted 65 minutes with an estimated blood loss of 50 mL. Gross examination of the resected specimen showed a left adrenal mass measuring 9.5 × 6.5 × 4.5 cm. The cyst wall thickness ranged from 0.2 to 0.5 cm with a smooth serosal surface and rough inner lining. The cyst contained abundant calcified material of hard consistency (Fig. 2). Histopathological examination demonstrated adrenal cystic changes with extensive calcium salt deposition within the cystic cavity, consistent with adrenal calcification. The final diagnosis was idiopathic massive adrenal calcification. The patient's postoperative course was uneventful, and she was discharged on postoperative day 3. Comparison with preoperative CT (Fig. 3A) confirmed complete resection on postoperative imaging (Fig. 3B). During the 18-month follow-up period, there was no evidence of calcification recurrence or any symptoms.

## 3 Discussion

Pathological calcium deposition may occur in vascular systems, parenchymal organs, and neoplastic tissues, with its formation mechanism potentially closely associated with underlying pathological alterations, disease progression, or chronic course of disease.^3^^,^^4^ Current research data on adrenal calcification remain limited. Available evidence suggests it may secondary to various pathological processes including adrenal hemorrhage, infectious lesions, inflammatory responses, and neoplastic disorders.^5^^,^^6^ However, systematic investigations into the precise etiology, morphological characteristics, clinical manifestations, and prognostic outcomes of adrenal calcification remain inadequate.

A single-center retrospective study of 540 patients revealed that adrenal calcification is predominantly detected incidentally during imaging examinations, with idiopathic calcification being the most prevalent, followed by tumor-associated calcification, post-hemorrhagic calcification, and infiltration disease-related calcification.^7^ From a pathological perspective, adrenal lesions with calcification demonstrate significant heterogeneity.^8^ The study cohort by Bhargav^9^ suggested that pheochromocytomas, adrenal cysts, and myelolipomas are more prone to exhibit calcification features. Jun Dai's study on Chinese populations found calcification more frequently associated with benign lesions including adrenal cysts, myelolipomas, cortical adenomas, hemangiolymphangiomas, and schwannomas, though it was also observed in malignant conditions such as adrenocortical carcinoma, liposarcoma, and metastatic tumors.^1^

Idiopathic massive adrenal calcification represents an exceptionally rare clinical entity. A large-scale retrospective study (n = 5057) revealed that only 1.48 % (75 cases) of patients undergoing adrenalectomy exhibited calcified lesions, with a median maximum diameter of 4.2 cm.^10^ Through systematic PubMed retrieval, we identified that fewer than 3 cases of idiopathic massive adrenal calcification have been reported worldwide to date. Among these, the case reported by Zhiqiang Ji et al.(with 6-month follow-up) represented the largest documented volume.^11^ The present case demonstrates two distinctive characteristics: 1) The calcified adrenal volume significantly exceeds all previously reported cases (preoperative CT showed a maximum diameter of 8.5 cm, with postoperative measurements of 9.5 × 6.5 × 4.5 cm); 2) The 18-month follow-up period represents the longest documented surveillance duration for this condition. The pathogenesis of idiopathic adrenal calcification remains incompletely understood, with several hypotheses currently proposed. Zhiqiang Ji et al. suggested potential associations with endocrine hormonal disturbances or vascular abnormalities.^11^ We postulate that the underlying mechanisms may involve: 1) tissue degeneration (calcific deposition after subclinical hemorrhage/infarction); 2) localized calcium-phosphorus metabolic dysregulation; and 3) matrix differentiation disorders secondary to embryonic developmental abnormalities. Notably, most affected patients lack typical clinical symptoms, with the condition predominantly detected incidentally through imaging studies. The precise pathophysiological mechanisms warrant further elucidation through multicenter case integration and molecular biological investigations.

Regarding the surgical indications for calcified adrenal lesions, no consensus has been reached in the academic community. According to current clinical guidelines, a conservative strategy involving regular imaging follow-up is recommended for adrenal lesions <3 cm in diameter without malignant features or clinical symptoms. The study by Bin et al. demonstrated that when the lesion diameter reaches ≥6 cm, the malignant potential increases significantly, and surgical intervention is clearly indicated.^8^ Notably, in cases complicated by hypertension or lumbar pain, the potential mechanisms of postoperative symptom relief may involve psychological alleviation, decompression of tumor compression on normal adrenal tissue, and reduction of mechanical compression on surrounding organs.^12^ In terms of surgical approach selection, a comprehensive evaluation of the lesion's anatomical characteristics (size, location) and the surgeon's technical expertise is required. Our institutional clinical practice has demonstrated that the retroperitoneal approach offers the following advantages: 1) avoidance of interference from abdominal organs; 2) improved surgical field exposure; 3) significant reduction in intraoperative blood loss; and 4) shorter operative time. However, for the large calcified lesions (>8 cm in diameter) involved in this study, special attention should be paid to the inherent limitations of the retroperitoneal approach—namely, restricted operative space. Therefore, we recommend that the procedure be performed by a surgical team with extensive experience in retroperitoneal surgery.

## CRediT authorship contribution statement

**Zaiqing Jiang:** Writing – original draft, Writing – review & editing. **Yunfeng Li:** Writing – review & editing. **Kai Zhao:** Data curation. **Xinbao Yin:** Data curation. **Ke Wang:** Supervision. **Zongliang Zhang:** Supervision.

## Ethics statement

The study involving human participant were reviewed and approved by The Affiliated Hospital of Qingdao University. Clinical trial number: not applicable.

## Consent for publication

Written informed consent for publication was obtained from the patient. Consent to Publish declaration: YES.

## Funding

This work was supported by the 10.13039/501100001809National Natural Science Foundation of China (No. 31971191 and No. 82200759).

## Conflict of interest

The authors had no conflict-of-interest to declare that are relevant to the content of this article.

## Data Availability

The de-identified data will be shared on reasonable request to the corresponding author.
